# The Value of Myocardial Torsion and Aneurysm Volume for Evaluating Cardiac Function in Rabbit with Left Ventricular Aneurysm

**DOI:** 10.1371/journal.pone.0121876

**Published:** 2015-04-09

**Authors:** Zhai Hong, Mu Yuming, Wang Chunmei, Yan Xue

**Affiliations:** 1 Department of Ultrasonography, Affiliated Traditional Chinese Medicine Hospital of Xinjiang Medical University, Urumqi, Xinjiang Uygur Autonomous Region, China; 2 Department of Echocardiography, First Affiliated Hospital, Xinjiang Medical University, Urumqi, Xinjiang Uygur Autonomous Region, China; University of Buenos Aires, Faculty of Medicine. Cardiovascular Pathophysiology Institute., ARGENTINA

## Abstract

**Objective:**

This study aimed to investigate the effect of left ventricular aneurysm (LVA) volume and left ventricular global torsion on cardiac function by real time three-dimensional echocardiography (RT-3DE) and two-dimensional speckle tracking imaging(2D-STI), to determine the accuracy of RT-3DE and 2D-STI in assessing LV function.

**Methods:**

Thirty New Zealand rabbit models of with LVA were prepared by ligation of the middle segment of the left anterior descending and left circumflex arteries. Four weeks post-procedure, RT-3DE was conducted to obtain data on LVEF, left ventricular end-diastolic volume (LVEDV), left ventricular end-systolic volume (LVESV), and LVA volume (LVAV), Peak rotation angles at the mitral valve annulus level (MV-ROT), peak rotation angles at the apical level (AP-ROT), and left ventricular global torsion angles (LV-TOR) were measured by 2D-STI.

**Results:**

Compared with controls, LVEDV and LVESV were significantly increased in the LVA group, while LVEF, MV-ROT, AP-ROT, and LV-TOR were consistently reduced (*p*<0.01). Moreover, LVEF correlated with LVA volume and LV torsion angle (r= -0.778 and 0.821, *p*<0.01). LVA volume/LVEDV had the strongest inverse relationship with LVEF (*r*= -0.911, *p*<0.01).

**Conclusion:**

LVA volume, LVA volume/LVEDV, and LV torsion may be used as an indicator for evaluation of cardiac function after LVA. Moreover, LVA volume/LVEDV may be a more sensitive and reliable marker of cardiac function after LVA formation.

## Introduction

Left ventricular aneurysm (LVA), one of the most important complications of myocardial infarction (MI), was defined as expansion of the dyskinetic area of the left ventricular wall. Aneurysms usually arise from a portion of weakened tissue in the ventricular wall, resulting in a reduction of the left ventricular ejection fraction (LVEF) [1–2].

Recently, assessing cardiac function has been addressed using real time three-dimensional echocardiography (RT-3DE) [3]. The RT-3DE technique does not depend on the geometry hypothesis for calculating left ventricular volume, especially when there is a discrepancy in cardiac anatomy (i.e. ventricular aneurysm) and is more accurate than two-dimensional echocardiography. RT-3DE is highly accurate and dependable for measuring left ventricular function [4–6], and correlate well with cardiac magnetic resonance imaging [7–10]. Prakash [11] demonstrated that RT-3DE accurately predicted volumes of a variety of asymmetric ventricular cavities.

The recent development of two-dimensional speckle tracking imaging (2D-STI) may also provide another means of assessing left ventricular function [12–13]. 2D-STI automatically tracks myocardial tissue signals, and can calculate myocardial tissue velocity, displacement, strain, strain rate, rotation angle, and myocardial mechanical parameters [14], as well as measure local and global myocardial deformation characteristics and cardiac rotation movement. Thus, such imaging modalities are of major clinical significance in myocardial ischemia, and measurement of cardiac function, cardiac torsion, and the cardiac synchronicity [15–17].

Previous research has shown that the development of LVA closely correlated with deterioration of cardiac function [18–19], as LVA development reduced the percentage of functioning myocardium, which contributed to LV ejection. Meanwhile, the aneurysm does not empty during systole (and may expand somewhat), so LV ejection of blood is less efficient, which leads to the decline of LVEF. At present, according to LVA size and its effect on cardiac function, different surgical approaches were chosen in clinical practice. Small LVA did not require to special treatment, while large LVAs or those with severe heart failure would require surgical treatment. The goal was to correct the size and geometry of the LV to reduce wall tension and paradoxical movement and to improve systolic function. If LVA volume could be measured accurately by RT-3DE and the effect of LVA volume on cardiac function could be evaluated accurately, this could aid in the choosing particular surgical approach in clinical treatment. However, the effect of LVA volume on cardiac function after LVA formation was rarely reported.

In addition, left ventricular torsion plays an important role in LV ejection and filling [20–21]; it is well established that LV torsion is sensitive to changes in both regional and global LV function [22–23]. Therefore, the assessment of LV torsion is a unique approach for quantifying LV function. Global LV torsion post MI was positively correlated with cardiac systolic function using 2D-STI [24]. However, the effect of LV torsion on cardiac function after LVA formation has not been previously reported.

Thus, we hypothesize that LVA volume and LV torsion may be more powerful variables for evaluating cardiac function after LVA formation. We aimed to investigate the relationship between cardiac function, LVA volume, and LV torsion using RT-3DE and 2D-STI, to determine the accuracy of RT-3DE and 2D-STI in assessing LV function, as well as to address the correlation between these echocardiographic variables and left ventricular function in the rabbit model, thus providing some useful parameters for clinical treatment of LVA.

## Methods

### Experimental animals and preparation of LVA models

30 New Zealand rabbits of both genders (weight 2.3–2.5 kg) were provided by the Experimental Animal Center of Xinjiang Medical University (Urumqi, Xinjiang, China). Rabbits were anesthetized with pentobarbitone sodium (30 mg/kg IV). Rabbit models of LVA were prepared by ligation of the middle segment of the left anterior descending artery and left circumflex artery, as previously described [25]. Preoperative intravenous lidocaine was administered to prevent arrhythmia and intravenous heparin was used for prophylaxis of venous thrombosis. Briefly, a thoracotomy was performed at the fourth left intercostal space and the left anterior descending and left circumflex arteries were permanently ligated in the middle segment with a 5–0 silk suture. Control animals underwent sham thoracotomy (n = 10).

All animal experiments were performed in strict accordance with the National Institutes of Health Guide for the Care and Use of Laboratory Animals (NIH Pub. No. 86–23, revised 1996) and with the approval of the Animal Care Committee of the First Affiliated Hospital, Xinjiang Medical University, P.R. China.

### RT-3DE image acquisition and analysis

In order to observe LVA formation and measure LVA volume, RT-3DE was used at postoperative days 1, 2 and 3, and weeks 1, 2, 3, and 4. This study found that the LVA of most animals were completely formed at postoperative 4 week and the LVA volume was the largest at postoperative 4 week. So the rabbits which had formed LVA were enrolled in this study at postoperative 4 week. The criteria for determination of LVA by echocardiography were: 1) marked thinning of the ventricular wall at the MI zone, with outward bulging of the ventricular wall in both systolic and diastolic phases; 2) the bulging wall displayed conflicting motion.

Four weeks postoperatively, the rabbits were re-anesthetized and echocardiography was performed using the iE33 dimension system (Philip, Inc, Bothell, WA, USA), with an X3-1 probe(volume rate was 24 Hz). RT-3DE images were acquired and left ventricular end-systolic and end-diastolic volume (LVESV and VLVEDV) and LVEF were calculated as described in our previous study [3]. LVA volume was measured and calculated as previously described [26]. Briefly, QLAB software GI 3DQ was activated and the vertical distance of the LVA (cranial-caudad) was marked. The software automatically divided the LVA into 9 adjustable cross-sections along the vertical plane. The outline of the LVA was depicted at the corresponding short axis cross-sections and the software would subsequently calculate LVA volume. The LVA volume to LVEDV ratio was manually calculated. Analysis of images was performed with QLAB analysis software (Philips, Andover, MA, USA). All measurements were averaged over five consecutive cardiac cycles.

### 2D-STI analysis

Two-dimensional echocardiography and 2D-STI studies were performed using the iE33 dimension system (Philip, Inc, Bothell, WA, USA), equipped with an S12-4 probe(frame rate was 70–90 Hz). The parasternal mitral valve annulus and apical short-axis view were used for measurement of the mitral valve annulus level and apical level rotation. The QLAB software automatically segmented each short axis of the LV into 6 segments (anterior septum, anterior wall, lateral wall, posterior wall, inferior wall, and inferior septum). LV rotation at the mitral valve level (MV-ROT) and at the apical level (AP-ROT) in each segment were calculated, as previously described [27]. The net difference in rotation angles at the two short-axis levels was defined as the global LV torsion angle. Analysis of images was performed with QLAB analysis software. All measurements were averaged for five consecutive cardiac cycles.

### Statistical analysis

Continuous variables were presented as the mean and standard deviation. Statistical comparisons of echocardiographic variables between the LVA and control groups were made by analysis of variance with the Student’s t-test. Correlation coefficients between LVEF and LVA volume, and LV torsion angles were calculated by linear regression analysis. A p-value of less than 0.05 was considered statistically significant. All data analyses were performed by SPSS 11.5 software (SPSS, In., Chicago, USA).

## Results

### LVA animal models evaluation

**Fig 1:**
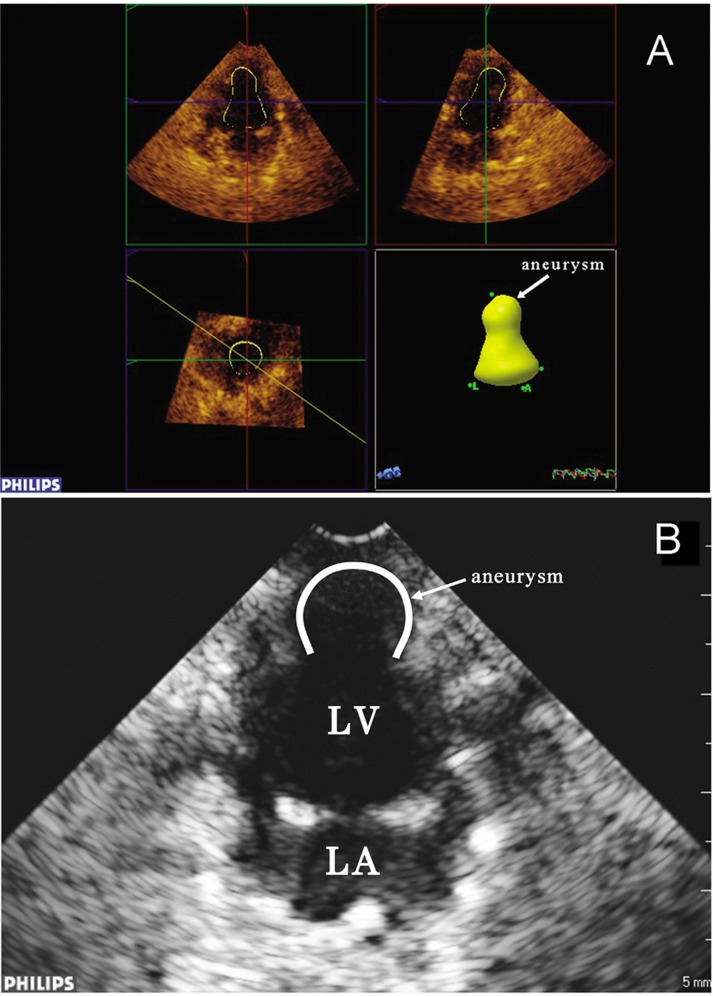
The diagnosis of LV aneurysm confirmed by the RT-3DE and2DE technique. (A: Marked arrow in the cardiac apex shows left ventricular aneurysm formation. B: Marked arrow in the cardiac apex shows left ventricular aneurysm formation at 2-chamber view).

Of the 30 New Zealand rabbits in the experimental group, 4 died within 4 weeks and 26 survived. Of these survived, 20 developed LVA and 6 did not (Fig 1, S1 Fig). No deaths occurred in the control group.

### Intergroup comparisons of echocardiographic variables

Compared with controls, LV volume was significantly increased at both the end-systolic and end-diastolic phase in the LVA group (LVESV 1.04±0.31 vs.; 2.32±0.52 LVEDV 2.85±0.34 vs.; 3.81±0.69 p<0.01). Conversely, the LVA group had a lower LVEF than the control group (38.72±6.86 vs. 63.12±6.36, p<0.01), (Table 1, S1 Table).

**Table 1:** Intergroup comparisons in left ventricle echocardiographic variables (mean ± standard deviation).

Group	n	LVEDV (ml)	LVESV (ml)	LVEF(%)	LVAV (ml)	LVAV/LVEDV(%)
Control	10	2.75±0.34	1.04±0.31	63.12±6.36	—	—
LVA	20	3.81±0.69	2.32±0.52	38.72±6.86	1.35±0.42	26.28±4.80
P values		<0.01	<0.01	<0.01	—	—

**Note:** LVEDV: left ventricular end-diastolic volume; LVESV: left ventricular end-diastolic volume; LVEF: left ventricular ejection fraction; LVAV: left ventricular aneurysm volume.

### Intergroup comparisons of left ventricular torsion angles

Compared with controls, the rotation angles of the 6 segments of the LV short-axis were significantly reduced in the LVA group. At the MV level, anterior septal rotation: -0.89±0.42° vs. -2.37±1.26°; anterior rotation: -0.37±0.08° vs. -2.03±1.01°; lateral rotation: -0.42±0.18° vs. -2.25±1.13°; posterior rotation: -0.75±0.26° vs. -2.81±1.52°; inferior rotation: -0.91±0.45° vs. -3.45±1.72°; inferior septal rotation: -0.73±0.33° vs. -3.34±1.15°. At the AP level, anterior septal rotation: 0.58±0.31° vs. 2.67±1.66°; anterior rotation: 0.22±0.22° vs. 2.32±1.11°; lateral rotation: 0.27±0.18° vs. 2.43±1.47°; posterior rotation: 0.51±0.46° vs. 3.65±1.42°; inferior rotation: 0.68±0.35° vs. 4.21±1.72°; inferior septal rotation: 0.60±0.37° vs. 3.12±1.15°. (all p<0.01), (Table 2, S2 Table).

**Table 2:** Intergroup comparisons in 6 segments rotation angles of left ventricle (°, mean ± standard deviation).

group	n	Anterior sept rotation	Anterior rotation	Lateral rotation	Posterior rotation	Inferior rotation	Inferior sept rotation
MV level							
control	10	-2.37±1.26	-2.03±1.01	-2.25±1.13	-2.81±1.52	-3.45±1.72	-3.34±1.15
LVA	20	-0.89±0.42	-0.37±0.08	-0.42±0.18	-0.75±0.26	-0.91±0.45	-0.73±0.33
P values		0.000	0.000	0.000	0.000	0.000	0.000
AP level							
control	10	2.67±1.66	2.32±1.11	2.43±1.47	3.65±1.42	4.21±1.72	3.12±1.15
LVA	20	0.58±0.31	0.22±0.22	0.27±0.18	0.51±0.46	0.68±0.35	0.60±0.37
P values		0.000	0.000	0.000	0.000	0.000	0.000

**Note:** MV level: at mitral valve annulus level; AP level: at apical level; Anterior sept rotation: the rotation angle of anterior septum; Anterior rotation: the rotation angle of anterior wall; Lateral rotation: the rotation angle of lateral wall; Posterior rotation: the rotation angle of posterior wall; Inferior rotation:the rotation angle of inferior wall; Inferior sept rotation:the rotation angle of inferior septum wall.

Compared with controls, the torsion angle of the 6 segments of the LV short-axis were significantly reduced in the LVA group(anterior septal torsion: 0.95±0.12° vs. 4.91±1.31°; anterior torsion: 0.48±0.13° vs. 4.32±1.77°; lateral torsion: 0.73±0.31° vs. 4.72±1.10°; posterior torsion: 1.08±0.16° vs. 5.02±1.41°; inferior torsion: 1.70±0.65° vs. 7.51±1.23°; inferior septal torsion: 1.37±0.51° vs. 6.42±1.06°,all p<0.01), (Table 3, S3 Table).

**Table 3:** Intergroup comparisons in 6 segments torsion angle (°, mean ± standard deviation).

group	n	Anterior sept TOR	Anterior TOR	Lateral TOR	Posterior TOR	Inferior TOR	Inferior sept TOR
control	10	4.91±1.31	4.32±1.77	4.72±1.10	5.02±1.41	7.51±1.23	6.42±1.06
LVA	20	0.95±0.12	0.48±0.13	0.73±0.31	1.08±0.16	1.70±0.65	1.37±0.51
P values		0.000	0.000	0.000	0.000	0.000	0.000

**Note:** Anterior sept TOR: the torsion angle of anterior septum; Anterio TOR: the torsion angle of anterior wall; Lateral TOR: the torsion angle of lateral wall;Posterior TOR: the torsion angle of posterior wall; Inferior TOR: the torsion angle of inferior wall; Inferior sept TOR:the torsion angle of inferior septum wall.

**Fig 2:**
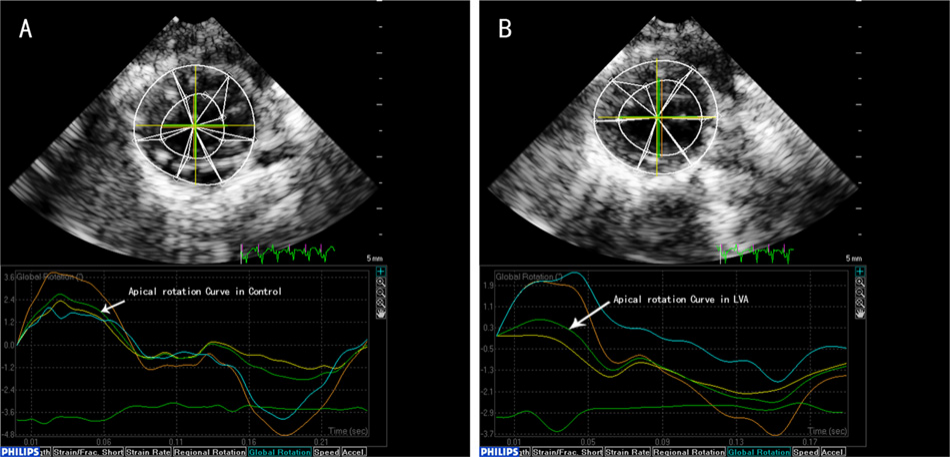
Apical rotation curves in control and LVA groups. (A:Green curve of the marked arrow shows the apical rotation curve in control group at left ventricular short-axis view. B: Green curve of the marked arrow shows the apical rotation decrease in LVA group at left ventricular short-axis view).

Compared with controls, the LV global rotation angle at the mitral valve annulus (MV-ROT), the LV global rotation angle at the apical level (AP-ROT), and the LV global torsion angle (LV-TOR) were significantly reduced in the LVA group, especially at the apical level (AP-ROT: 0.45±0.21° vs. 2.85±1.10°, MV-ROT: -0.92±0.11° vs. -2.58±1.26°, LV-TOR: 1.05±0.32° vs. 4.65±1.50°, all p<0.01), (Table 4, Fig 2, S4 Table, S2 Fig).

**Table 4:** Intergroup comparisons in left ventricle torsion angles (°, mean ± standard deviation).

Group	n	AP-ROT(°)	MV-ROT(°)	LV-TOR(°)
Control	10	2.85±1.10	-2.58±1.26	4.65±1.50
LVA	20	0.45±0.21	-0.92±0.11	1.05±0.32
P values		<0.01	<0.01	<0.01

**Note:** MV-ROT: peak rotation angle at mitral valve annulus level; AP-ROT: peak rotation angle at apical level; LV-ROT: left ventricular global rotation angle.

### Correlation between LVEF and LVEDV, LVA volume, LV-TOR

**Fig 3:**
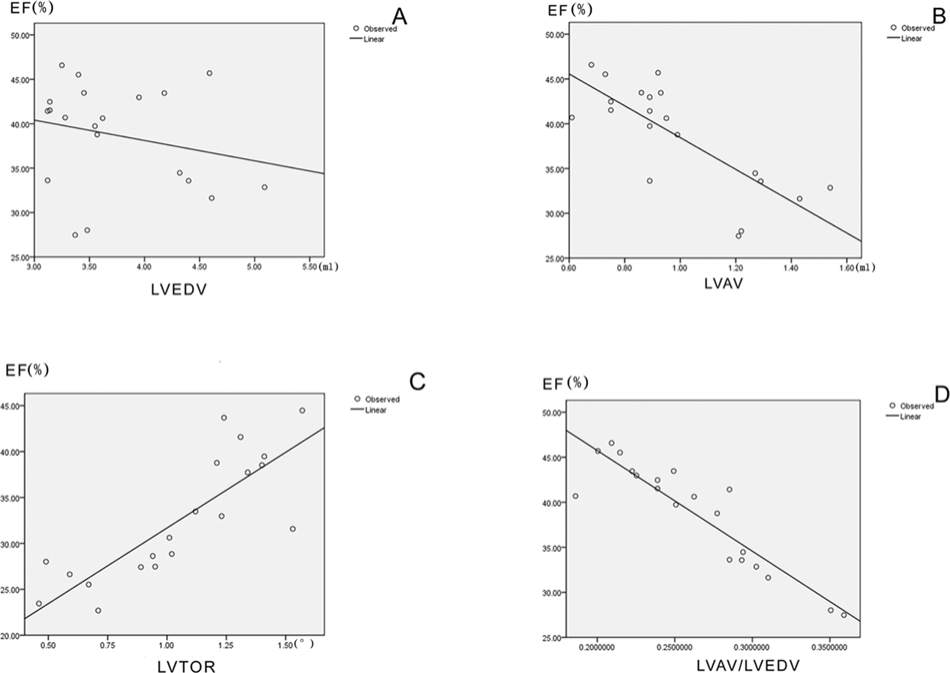
Correlations between LVEF and LVEDV, LVAV, LV-ROT and LVAV/LVEDV. (A:Correlation between LVEF and LVEDV, r = -0.232, p = 0.324, B:Correlation between LVEF and LVAV, r = -0.778, p = 0.000, C: Correlation between LVEF and LV-ROT, r = 0.821, p = 0.000, D:Correlation between LVEF and LVAV/LVEDV,r = -0.911,p = 0.000).)

Significant correlations between LVEF and LVA volume, LV-TOR, LVAV volume/LVEDV were observed (r = -0.778, 0.821, and -0.911, respectively, p<0.01), (Fig 3, S3 Fig). However, the correlation between LVEF and LVEDV was not found to be statistically significant (r = -0.232, p = 0.324). Of note, the ratio of LVAV to LVEDV had the tightest inverse correlation with LVEF.

## Discussion

This study found that the development of LVA was associated with an increase in LVEDV and LVESV, and a reduction in LVEF. Meanwhile, there was a significant reduction in LV global rotation and torsion angle in all segments. Moreover, LVEF and LVA volume, LV global torsion, and LVAV/LVEDV strongly correlated. The LVA volume/LVEDV ratio presented the tightest inverse relationship with LVEF.

Our study confirmed findings of previous investigations that LVA formation, as a complication of MI, can be attributed to left ventricular remodeling [28]. Unfavorable remodeling and LVA formation results in an increase in LV pressure-load and volume-load as well as LV enlargement, subsequently leading to low cardiac output and an accelerated development of cardiac insufficiency and subsequent deterioration of cardiac function [29].

Previous studies have consistently shown an impairment in LV torsional deformation in the setting of acute and chronic MI [30–31]. However, there is a paucity of data on the changes in LV torsion after LVA. We demonstrated a significant decline in the torsion angles and global torsion angles in all segments. Prior research has shown that epicardial myofibers are important for maintaining LV torsional deformation and to determine the overall direction of rotation [32]. In addition, LV myofibers have a typical spiral architecture that is also important in determining LV systolic wringing motion [33]. LVA development is based upon transmural myocardial infarction, and subsequent myocardial necrosis and fibrosis, which reduces the amount of functioning myocardium contributing to LV ejection, leading to distortion of LV myofiber architecture, altering the oblique direction and eventually impairing LV torsion.

Recently, the prognostic value of the LV volume in assessing cardiac function was addressed using RT-3DE [1–3]. However, the high correlation between LVEDV and LVEF was not present in our study. Perhaps this is because LVA formation led to a change in LV geometry, which reduced the effect of LVEDV on cardiac function. LV torsion is also important and sensitive for LV systolic function [34]. Our study confirmed previous observations. Moreover, LVA volume had a higher correlation with LVEF. Specifically, the correlation between the LVAvolume to LVEDV ratio and LVEF was higher than that between LVEF, LVA volume, and LV torsion. According to the mechanical theory, LVA volume reflects the distribution and range of injured myocardium. On the one hand, conflicting motion in the LVA region reduces global LV contractility and synchronization; on the other hand, LVA volume also influences left ventricular volume-load, so that LVA volume may be a useful parameter for evaluating cardiac function. Meanwhile the ratio of LVA volume to LVEDV to some degree reflects cardiac output; the higher the ratio of LVA volume to LVEDV, the lower the cardiac output, because the aneurysm does not empty during systole and may expand somewhat. Thus, LV ejection is less efficient, which leads to a decline in LVEF. The major superiority is the high homogeneity in LVA volume to LVEDV ratio, and considerable heterogeneity in LVA volume and LVEDV among individuals. This finding suggests that the LVA volume /LVEDV ratio may be more sensitive than LVA volume and LVEDV in evaluating cardiac function, with LVA occurring secondary to myocardial infarction.

## Study Limitations

This study had several limitations. The first, our sample size was small so we were not able to make continuous observations of the process of ventricular aneurysm formation, In future studies, we plan to increase sample size in subsequent studies. Second, the invasive measurements of cardiac output and LV pressure were not performed, and some parameters, such as LV volume and LVEF by 3DE were not compared with other results by magnetic resonance tomography in the present study. In this study, the term “torsion” is applied to mean the net difference between the rotation of the base and the apex. However, more correctly, the net difference should be indexed by the length of the ventricle, which was not done in this study.

## Conclusion

This study demonstrated that LVA volume on RT-3DE and LV torsion on 2D-STI are closely associated with LVEF. Accordingly, these parameters may be used in clinical practice as an indicator for the evaluation of cardiac function after LVA. Moreover, LVA volume/LVEDV measured with RT-3DE may be more sensitive and reliable than LVA volume and LV torsion in evaluating cardiac function after myocardial infarction followed by LVA.

## Supporting Information

S1 Fig The diagnosis of LV aneurysm confirmed by the RT-3DE (A) and 2DE technique (B). A: Marked arrow in the cardiac apex shows left ventricular aneurysm formation. B: Marked arrow in the cardiac apex shows left ventricular aneurysm formation at 2-chamber view.
(DOC)

S2 Fig Apical rotation curves in control and LVA groups. A: Green curve of the marked arrow shows the apical rotation curve in control group at left ventricular short-axis view. B: Green curve of the marked arrow shows the apical rotation decrease in LVA group at left ventricular short-axis view.
(DOC)

S3 Fig Correlations between LVEF and LVEDV, LVAV, LV-ROT and LVAV/LVEDV. A: Correlation between LVEF and LVEDV(r = -0.232, p = 0.324), B:Correlation between LVEF and LVAV(r = -0.778, p = 0.000), C: Correlation between LVEF and LV-ROT(r = 0.821, p = 0.000),D:Correlation between LVEF and LVAV/LVEDV(r = -0.911,p = 0.000).
(DOC)

S1 Table Intergroup comparisons in left ventricle echocardiographic variables. LVEDV: left ventricular end-diastolic volume; LVESV: left ventricular end-diastolic volume; LVEF: left ventricular ejection fraction; LVAV: left ventricular aneurysm volume.
(DOC)

S2 Table Intergroup comparisons in 6 segments rotation angles of left ventricle. MV level: at mitral valve annulus level; AP level: at apical level; Anterior sept rotation: the rotation angle of anterior septum; Anterior rotation: the rotation angle of anterior wall; Lateral rotation: the rotation angle of lateral wall; Posterior rotation: the rotation angle of posterior wall; Inferior rotation:the rotation angle of inferior wall; Inferior sept rotation:the rotation angle of inferior septum wall.
(DOC)

S3 Table Intergroup comparisons in 6 segments torsion angle. Anterior sept TOR: the torsion angle of anterior septum; Anterio TOR: the torsion angle of anterior wall; Lateral TOR: the torsion angle of lateral wall;Posterior TOR: the torsion angle of posterior wall; Inferior TOR: the torsion angle of inferior wall; Inferior sept TOR:the torsion angle of inferior septum wall.
(DOC)

S4 Table Intergroup comparisons in left ventricle torsion angles. MV-ROT: peak rotation angle at mitral valve annulus level; AP-ROT: peak rotation angle at apical level; LV-ROT: left ventricular global rotation angle.
(DOC)
