# Supplementary material for: The Value of Myocardial Torsion and Aneurysm Volume for Evaluating Cardiac Function in Rabbit with Left Ventricular Aneurysm
Source: PLoS One. 2015 Apr 9;10(4):e0121876. doi: 10.1371/journal.pone.0121876 (PMC4391835; doi:10.1371/journal.pone.0121876)
Supplement: S1 Fig — A: Marked arrow in the cardiac apex shows left ventricular aneurysm formation. B: Marked arrow in the cardiac apex shows left ventricular aneurysm formation at 2-chamber view. (DOC) [file pone.0121876.s001.doc]

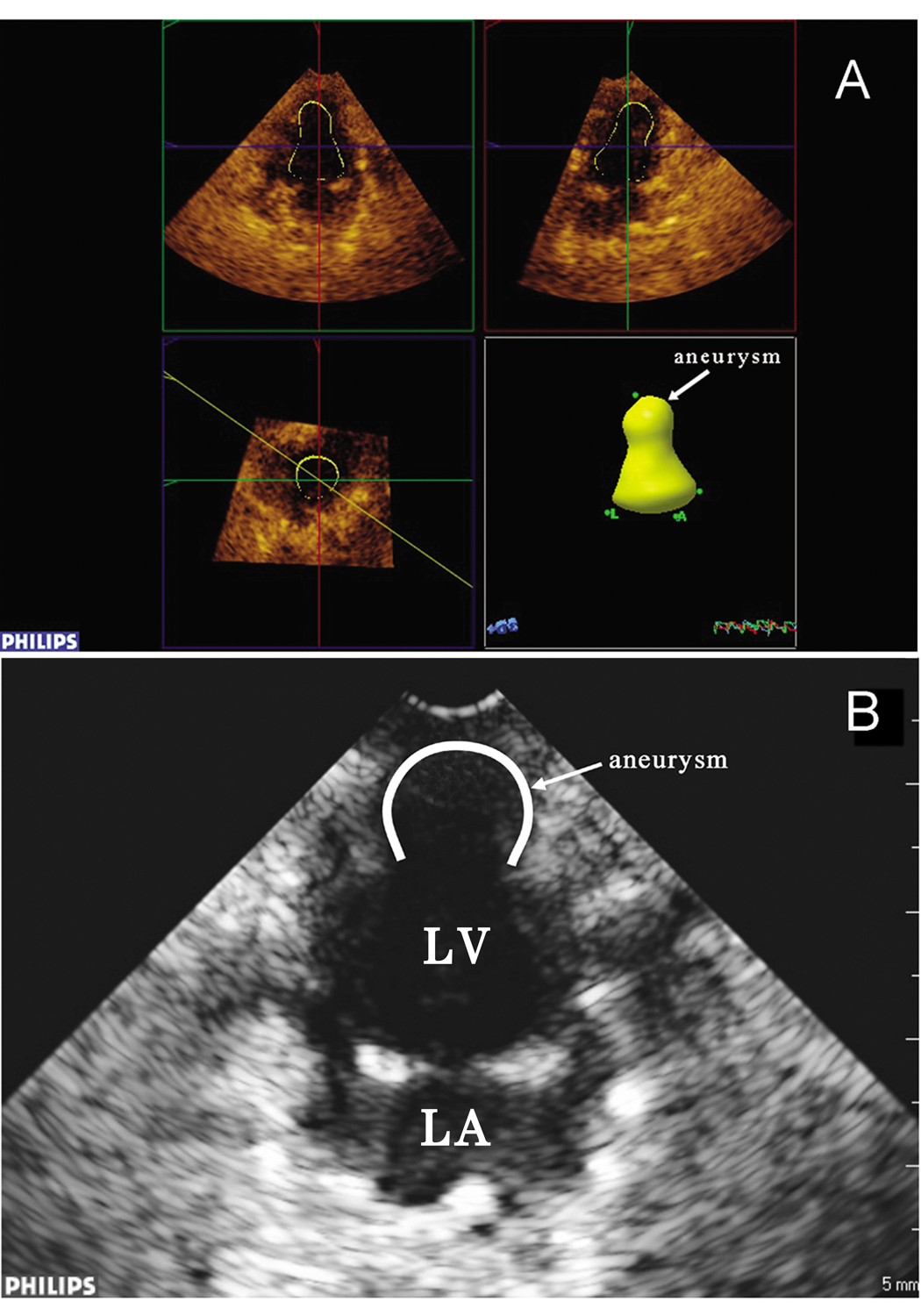


**Fig.1 The diagnosis of LV aneurysm confirmed by the RT-3DE (A) and 2DE technique(B).**

A: Marked arrow in the cardiac apex shows left ventricular aneurysm formation. B: Marked arrow in the cardiac apex shows left ventricular aneurysm formation at 2-chamber view .
