# Supplementary material for: The Value of Myocardial Torsion and Aneurysm Volume for Evaluating Cardiac Function in Rabbit with Left Ventricular Aneurysm
Source: PLoS One. 2015 Apr 9;10(4):e0121876. doi: 10.1371/journal.pone.0121876 (PMC4391835; doi:10.1371/journal.pone.0121876)
Supplement: S2 Fig — A: Green curve of the marked arrow shows the apical rotation curve in control group at left ventricular short-axis view. B: Green curve of the marked arrow shows the apical rotation decrease in LVA group at left ventricular short-axis view. (DOC) [file pone.0121876.s002.doc]

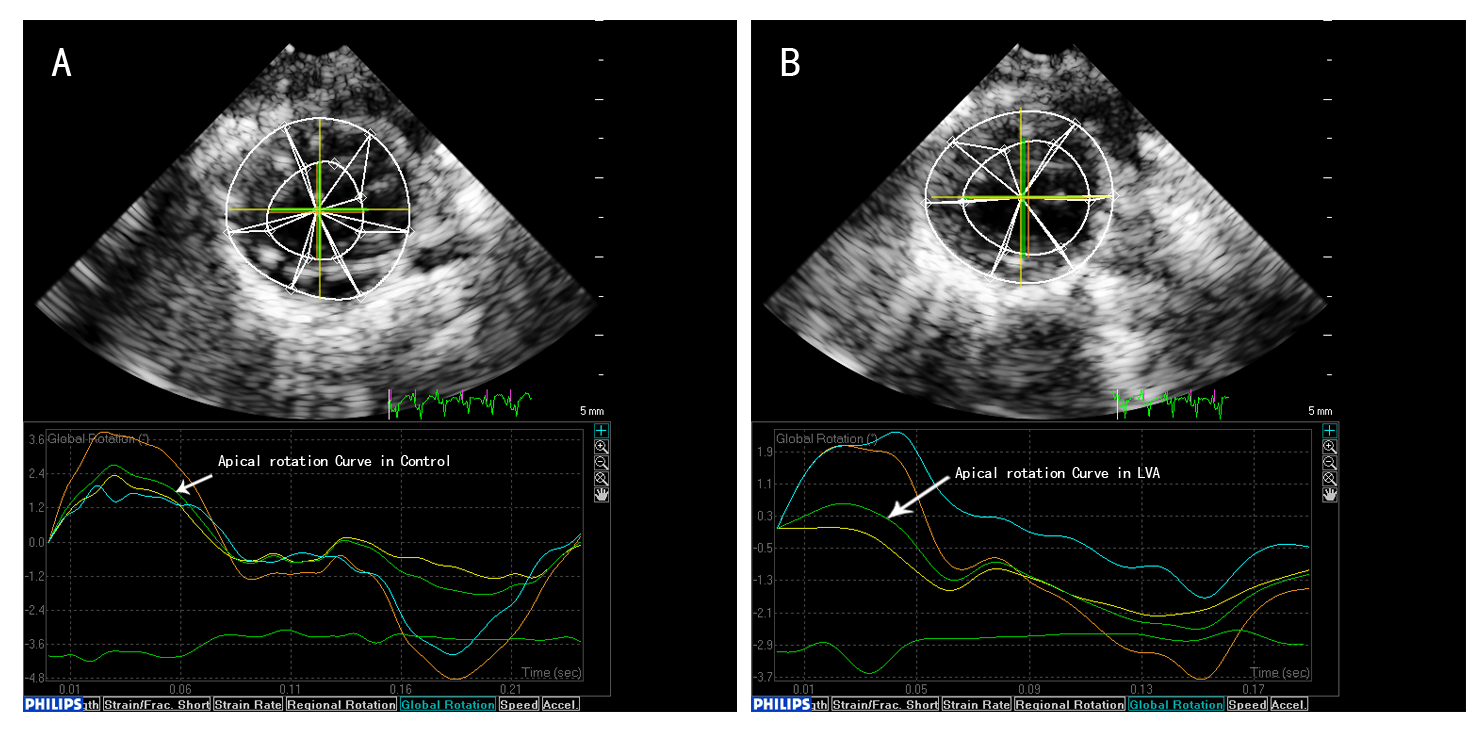


**Fig. 2 Apical rotation curves in control and LVA groups .**

A:Green curve of the marked arrow shows the apical rotation curve in control group at left ventricular short-axis view. B: Green curve of the marked arrow shows the apical rotation decrease in LVA group at left ventricular short-axis view .
