# Supplementary material for: The Value of Myocardial Torsion and Aneurysm Volume for Evaluating Cardiac Function in Rabbit with Left Ventricular Aneurysm
Source: PLoS One. 2015 Apr 9;10(4):e0121876. doi: 10.1371/journal.pone.0121876 (PMC4391835; doi:10.1371/journal.pone.0121876)
Supplement: S3 Fig — A: Correlation between LVEF and LVEDV(r = -0.232, p = 0.324), B:Correlation between LVEF and LVAV(r = -0.778, p = 0.000), C: Correlation between LVEF and LV-ROT(r = 0.821, p = 0.000),D:Correlation between LVEF and LVAV/LVEDV(r = -0.911,p = 0.000). (DOC) [file pone.0121876.s003.doc]

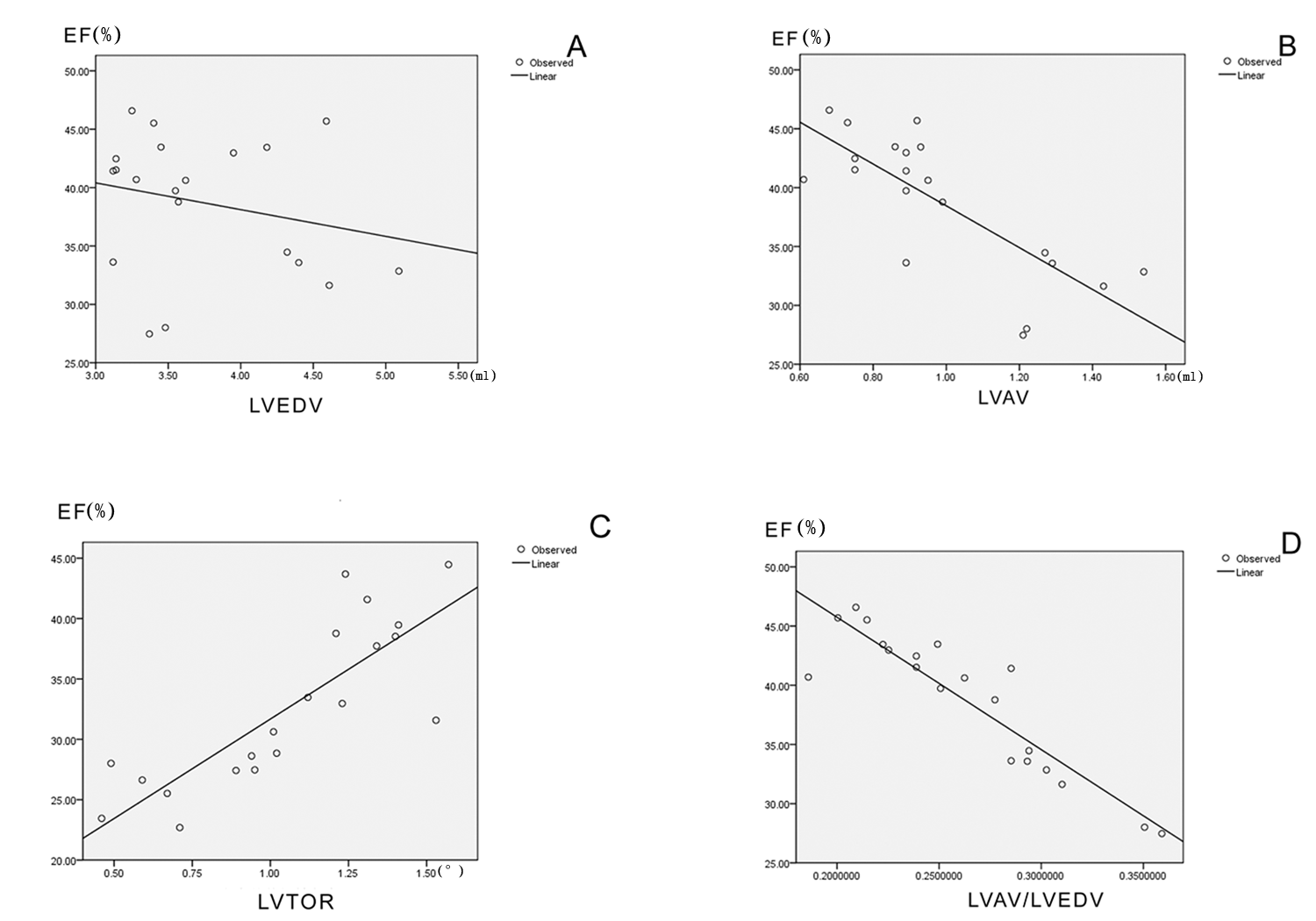


**Fig. 3 Correlations between LVEF and LVEDV, LVAV, LV-ROT and LVAV/LVEDV .**

A:Correlation between LVEF and LVEDV(r=-0.232, p=0.324), B:Correlation between LVEF and LVAV(r=-0.778, p=0.000), C: Correlation between LVEF and LV-ROT(r=0.821, p=0.000),D:Correlation between LVEF and LVAV/LVEDV(r=-0.911,p=0.000 ).
