# Supplementary material for: The Value of Myocardial Torsion and Aneurysm Volume for Evaluating Cardiac Function in Rabbit with Left Ventricular Aneurysm
Source: PLoS One. 2015 Apr 9;10(4):e0121876. doi: 10.1371/journal.pone.0121876 (PMC4391835; doi:10.1371/journal.pone.0121876)
Supplement: S1 Table — LVEDV: left ventricular end-diastolic volume; LVESV: left ventricular end-diastolic volume; LVEF: left ventricular ejection fraction; LVAV: left ventricular aneurysm volume. (DOC) [file pone.0121876.s004.doc]

**Table 1 Intergroup comparisons in left ventricle echocardiographic variables（mean ± standard deviation）**

| **Group** | **n** | **LVEDV (ml)** | **LVESV (ml)** | **LVEF(%)** | **LVAV（ml）** | **LVAV/LVEDV(%)** |
| --- | --- | --- | --- | --- | --- | --- |
| Control | 10 | 2.75±0.34 | 1.04±0.31 | 63.12±6.36 | — | — |
| LVA | 20 | 3.81±0.69 | 2.32±0.52 | 38.72±6.86 | 1.35±0.42 | 26.28±4.80 |
| P values | | <0.01 | <0.01 | <0.01 | — | — |

**Note :** LVEDV: left ventricular end-diastolic volume; LVESV: left ventricular end-diastolic volume; LVEF: left ventricular ejection fraction; LVAV：left ventricular aneurysm volume

**S1_Table .doc. Intergroup comparisons in left ventricle echocardiographic variables.**

LVEDV: left ventricular end-diastolic volume; LVESV: left ventricular end-diastolic volume; LVEF: left ventricular ejection fraction; LVAV：left ventricular aneurysm volume
