# Supplementary material for: The Value of Myocardial Torsion and Aneurysm Volume for Evaluating Cardiac Function in Rabbit with Left Ventricular Aneurysm
Source: PLoS One. 2015 Apr 9;10(4):e0121876. doi: 10.1371/journal.pone.0121876 (PMC4391835; doi:10.1371/journal.pone.0121876)
Supplement: S2 Table — MV level: at mitral valve annulus level; AP level: at apical level; Anterior sept rotation: the rotation angle of anterior septum; Anterior rotation: the rotation angle of anterior wall; Lateral rotation: the rotation angle of lateral wall; Posterior rotation: the rotation angle of posterior wall; Inferior rotation:the rotation angle of inferior wall; Inferior sept rotation:the rotation angle of inferior septum wall. (DOC) [file pone.0121876.s005.doc]

**Table 2 Intergroup comparisons in 6 segments rotation angles of left ventricle （°，mean ± standard deviation）**

| **group** | **n** | **Anterior sept rotation** | **Anterior rotation** | **Lateral rotation** | **Posterior rotation** | **Inferior rotation** | **Inferior sept rotation** |
| --- | --- | --- | --- | --- | --- | --- | --- |
|  |  |  |  | MV level |  |  |  |
| control | 10 | -2.37±1.26 | -2.03±1.01 | -2.25±1.13 | -2.81±1.52 | -3.45±1.72 | -3.34±1.15 |
| LVA | 20 | -0.89±0.42 | -0.37±0.08 | -0.42±0.18 | -0.75±0.26 | -0.91±0.45 | -0.73±0.33 |
| P values |  | 0.000 | 0.000 | 0.000 | 0.000 | 0.000 | 0.000 |
|  |  |  |  | AP level |  |  |  |
| control | 10 | 2.67±1.66 | 2.32±1.11 | 2.43±1.47 | 3.65±1.42 | 4.21±1.72 | 3.12±1.15 |
| LVA | 20 | 0.58±0.31 | 0.22±0.22 | 0.27±0.18 | 0.51±0.46 | 0.68±0.35 | 0.60±0.37 |
| P values |  | 0.000 | 0.000 | 0.000 | 0.000 | 0.000 | 0.000 |

**Note:** MV level: at mitral valve annulus level; AP level : at apical level; Anterior sept rotation: the rotation angle of anterior septum ; Anterior rotation: the rotation angle of anterior wall ; Lateral rotation: the rotation angle of lateral wall ;Posterior rotation: the rotation angle of posterior wall; Inferior rotation:the rotation angle of inferior wall ; Inferior sept rotation:the rotation angle of inferior septum wall .
