# Supplementary material for: The Value of Myocardial Torsion and Aneurysm Volume for Evaluating Cardiac Function in Rabbit with Left Ventricular Aneurysm
Source: PLoS One. 2015 Apr 9;10(4):e0121876. doi: 10.1371/journal.pone.0121876 (PMC4391835; doi:10.1371/journal.pone.0121876)
Supplement: S3 Table — Anterior sept TOR: the torsion angle of anterior septum; Anterio TOR: the torsion angle of anterior wall; Lateral TOR: the torsion angle of lateral wall;Posterior TOR: the torsion angle of posterior wall; Inferior TOR: the torsion angle of inferior wall; Inferior sept TOR:the torsion angle of inferior septum wall. (DOC) [file pone.0121876.s006.doc]

**Table 3 Intergroup comparisons in 6 segments torsion angle（°，mean ± standard deviation）**

| **group** | **n** | **Anterior sept TOR** | **Anterior TOR** | **Lateral TOR** | **Posterior TOR** | **Inferior TOR** | **Inferior sept TOR** |
| --- | --- | --- | --- | --- | --- | --- | --- |
| control | 10 | 4.91±1.31 | 4.32±1.77 | 4.72±1.10 | 5.02±1.41 | 7.51±1.23 | 6.42±1.06 |
| LVA | 20 | 0.95±0.12 | 0.48±0.13 | 0.73±0.31 | 1.08±0.16 | 1.70±0.65 | 1.37±0.51 |
| P values |  | 0.000 | 0.000 | 0.000 | 0.000 | 0.000 | 0.000 |

**Note:** Anterior sept TOR: the torsion angle of anterior septum ; Anterio TOR: the torsion angle of anterior wall; Lateral TOR: the torsion angle of lateral wall;Posterior TOR: the torsion angle of posterior wall; Inferior TOR : the torsion angle of inferior wall; Inferior sept TOR:the torsion angle of inferior septum wall.
