# Supplementary material for: The Value of Myocardial Torsion and Aneurysm Volume for Evaluating Cardiac Function in Rabbit with Left Ventricular Aneurysm
Source: PLoS One. 2015 Apr 9;10(4):e0121876. doi: 10.1371/journal.pone.0121876 (PMC4391835; doi:10.1371/journal.pone.0121876)
Supplement: S4 Table — MV-ROT: peak rotation angle at mitral valve annulus level; AP-ROT: peak rotation angle at apical level; LV-ROT: left ventricular global rotation angle. (DOC) [file pone.0121876.s007.doc]

**Table 4 Intergroup comparisons in left ventricle torsion angles（°，mean ± standard deviation**）

| **Group** | **n** | **AP-ROT(°)** | **MV-ROT(°)** | **LV-TOR(°)** |
| --- | --- | --- | --- | --- |
| Control | 10 | 2.85±1.10 | -2.58±1.26 | 4.65±1.50 |
| LVA | 20 | 0.45±0.21 | -0.92±0.11 | 1.05±0.32 |
| P values |  | <0.01 | <0.01 | <0.01 |

**Note:** MV-ROT：peak rotation angle at mitral valve annulus level；AP-ROT：

peak rotation angle at apical level；LV-ROT：left ventricular global rotation angle .
